# Rapidly Progressive Triple M Overlap Syndrome After Immune Checkpoint Inhibitor Therapy

**DOI:** 10.7759/cureus.112508

**Published:** 2026-07-12

**Authors:** Seth Garrett, Anam Ansari, Sruthi Siddada, Shobana Sankar, Amita Bishnoi, Alireza Meysami, Sufiya Hussain

**Affiliations:** 1 Internal Medicine, Henry Ford Health System, Detroit, USA; 2 Rheumatology, Henry Ford Health System, Detroit, USA; 3 Internal Medicine, Henry Ford Macomb Hospital, Clinton Township, USA

**Keywords:** immune checkpoint inhibitor, immune-related adverse event (irae), myasthenia-like syndrome, myocarditis, myositis, triple-m overlap syndrome

## Abstract

Immune checkpoint inhibitors (ICIs) are widely used in cancer treatment, but can cause immune-related adverse events affecting multiple organ systems. Triple M overlap syndrome (TMOS) is a rare and often fatal complication, consisting of the triad of ICI-mediated myositis, myocarditis, and myasthenia gravis.

An 85-year-old man with metastatic renal cell carcinoma was initiated on combination ICI-based therapy with pembrolizumab and lenvatinib. Approximately four weeks after initiation of immunotherapy, he presented to the emergency department following a two-week history of progressive neck extensor weakness, downward gaze, worsening proximal upper and lower extremity weakness, recurrent falls, dysphagia, and dysphonia. On evaluation, myositis, myocarditis, and clinical evidence of a myasthenia gravis-like syndrome were noted, and TMOS was diagnosed. Initial treatment with high-dose intravenous corticosteroids and intravenous immunoglobulin was started, yet the patient's condition deteriorated. Despite aggressive treatment, due to hemodynamic instability and liver injury, he could not be started on plasmapheresis or second-line immunosuppression. With progressive multiorgan failure, the patient’s management was shifted to comfort-based measures.

Although an increasing number of immunosuppressive agents have shown efficacy in TMOS management, this case illustrates how the multiorgan injury inherent to TMOS can itself create contraindications to the very therapies required for definitive treatment. This self-reinforcing cycle of therapeutic limitation highlights the importance of early recognition and aggressive management of TMOS prior to end-organ damage, narrowing the treatment window.

## Introduction

Immune checkpoint inhibitors (ICIs) have transformed cancer treatment, but can cause immune-related adverse events (irAEs) affecting virtually all organ systems [1,2]. Among the most severe is triple M overlap syndrome (TMOS), a rare and often fatal complication consisting of the triad of ICI-mediated myositis, myocarditis, and myasthenia gravis or myasthenia gravis-like syndrome [3-5]. TMOS occurs in less than 1% of patients receiving ICI therapy, yet carries an inpatient mortality rate of 40%-60%, significantly higher than that of dual overlap syndromes involving myocarditis with either myositis or myasthenia gravis alone [3,6,7]. Disease onset typically occurs within the first month of treatment, with a median onset of 24-28 days, and cardiac conduction abnormalities and respiratory failure from respiratory muscle involvement are the leading causes of death [3,5,7,8].

TMOS poses distinct diagnostic challenges. No single confirmatory test exists; rather, the diagnosis requires clinical recognition of the concurrent presence of features of all three component conditions in the setting of ICI use [7,9]. Myasthenia gravis antibody testing and early cardiac imaging may be normal or nondiagnostic early in the disease course, and clinical features of myositis and myasthenia gravis frequently overlap, particularly bulbar dysfunction, respiratory weakness, and proximal limb weakness, making it difficult to distinguish the relative contribution of each component [9,10]. Furthermore, traditional diagnostic tools, such as repetitive nerve stimulation, have limited sensitivity when restricted to distal muscles, and myositis-specific antibodies are characteristically absent in ICI-related myositis, unlike idiopathic inflammatory myopathies [5,9]. These diagnostic complexities, combined with the potential for rapid clinical deterioration, underscore the importance of early recognition and a low threshold for multisystem evaluation in any ICI-treated patient presenting with neuromuscular symptoms.

We report a fatal case of TMOS in an 85-year-old man with metastatic renal cell carcinoma treated with pembrolizumab and lenvatinib. To our knowledge, this case highlights an underrecognized clinical phenomenon in TMOS: a self-reinforcing cycle in which the multisystem organ injury inherent to the syndrome, compounded by hepatotoxicity from a tyrosine kinase inhibitor, progressively eliminated each available therapeutic option for escalation of immunosuppression.

## Case presentation

An 85-year-old man with a history of stage III (pT3a) left chromophobe renal cell carcinoma, status post left nephrectomy one year prior, was found to have new lymphadenopathy on routine oncologic surveillance, with biopsy confirming new metastatic disease, as shown in Figure 1. He was initiated on combination ICI-based therapy with pembrolizumab and lenvatinib. He received a single dose of pembrolizumab. Lenvatinib was subsequently withheld 13 days prior to emergency department (ED) presentation because of new-onset transaminitis, and the patient missed his second cycle of pembrolizumab after developing nonspecific malaise.

**Figure 1:**
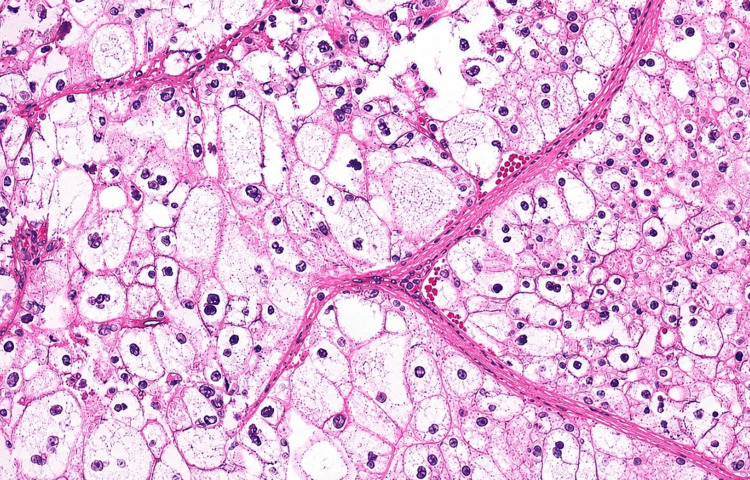
Biopsy section of abdominal lymph node Hematoxylin and eosin-stained section of metastatic renal cell carcinoma involving an abdominal lymph node, showing tumor cells with pale eosinophilic cytoplasm, irregular hyperchromatic nuclei, and fibrovascular septations.

Approximately four weeks after initiation of immunotherapy, he presented to the emergency department following a fall. His family reported a two-week history of progressive neck extensor weakness resulting in difficulty holding his head upright ("dropped head") and a downward gaze, accompanied by worsening proximal upper extremity weakness and recurrent falls, as well as new dysphagia and dysphonia.

On examination, vital signs were notable for blood pressure 123/61 mmHg, pulse 65 bpm, temperature 36.1 °C, respiratory rate 17 breaths per minute, and SpO₂ 98% on room air. The cervical spine was tender to palpation. Neurological examination revealed hypernasal speech with mild dysarthria. Cranial nerve examination demonstrated no ptosis, intact extraocular movements without nystagmus, intact facial sensation and symmetry, intact hearing bilaterally, midline uvula and palate elevation, midline tongue protrusion, but bilateral weakness of shoulder shrug (cranial nerve XI). Motor examination using the Medical Research Council (MRC) scale demonstrated neck flexor weakness (3/5), neck extensor weakness (2-3/5), bilateral deltoid weakness (5−/5), bilateral biceps weakness (4+/5), and bilateral triceps weakness (5−/5), with preserved distal upper extremity strength (5/5) and full lower extremity strength throughout (5/5). The patient was unable to hold his head upright in the sitting position and could not extend his head to neutral or control it eccentrically. Sensory examination revealed no dermatomal or distal-to-proximal gradient impairments. Deep tendon reflexes were 1+ at the brachioradialis, biceps, and triceps bilaterally, 2+ at the quadriceps bilaterally, and 1+ at the ankles bilaterally. Coordination testing was limited by difficulty lifting the arms rather than true ataxia. Formal bedside fatigability testing, including sustained upgaze for ptosis, repetitive proximal muscle testing (e.g., sustained shoulder abduction or repetitive deltoid testing against resistance), and timed assessments of sustained posture, was not performed at the time of initial evaluation. This was due to the severity of the patient's baseline weakness (neck extensors 2-3/5, neck flexors 3/5), which precluded meaningful assessment of fatigable decrement, as well as the clinical urgency of the presentation. The patient additionally endorsed muscle pain and reported dysphagia with emesis through the nares and food becoming stuck.

On evaluation, laboratory studies revealed markedly elevated creatine kinase (CK), elevated transaminases, and markedly elevated high-sensitivity cardiac troponin I, with results shown in Table 1. Transaminitis was attributed to drug-induced liver injury from lenvatinib, which had prompted its earlier discontinuation; notably, transaminitis was worsening at the time of ED presentation despite lenvatinib having been held for 13 days. Magnetic resonance imaging (MRI) of the cervical spine demonstrated extensive edema of the posterior cervical paraspinal musculature, as shown in Figure 2. Cardiac monitoring showed alternating bundle branch block with intermittent high-grade atrioventricular block with ventricular escape rhythm requiring temporary transvenous cardiac pacing. Following resolution of the atrioventricular block, he developed atrial fibrillation with rapid ventricular response requiring antiarrhythmic therapy.

**Table 1:** Laboratory results upon initial emergency department presentation ALT: alanine aminotransferase; AST: aspartate aminotransferase Note: Lactate dehydrogenase (LDH), aldolase, erythrocyte sedimentation rate (ESR), C-reactive protein (CRP), and ferritin were not obtained on admission.

Laboratory tests	Values	Normal range
Hemoglobin	15.2	13.5 - 17.0 g/dL
White blood cell count	12.0	3.8 - 10.6 x 10⁹/L
Neutrophils	10.5	1.8 - 7.70 x 10⁹/L
Platelets	331.0	150 - 450 x 10⁹/L
Urea	75	10 - 25 mg/dL
Creatinine	1.78	<1.13 mg/dL
Total bilirubin	1.3	<1.2 mg/dL
ALT	800	<52 IU/L
AST	560	<45 IU/L
Creatine kinase	6287	<250 IU/L
High-sensitivity cardiac troponin I	5241	<19 ng/L

**Figure 2:**
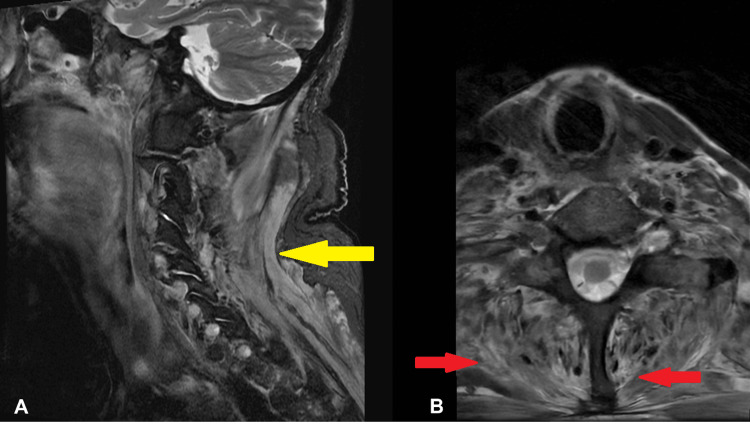
Cervical spine MRI Cervical spine MRI with sagittal (A) and axial (B) T2-weighted fat-suppressed MRI sequences are shown, demonstrating extensive bilateral hyperintense signal within the posterior cervical paraspinal musculature, consistent with diffuse inflammatory myositis. The yellow arrow indicates a T2-hyperintense signal of the posterior cervical paraspinal musculature within the sagittal view, while the red arrows indicate the bilateral T2-hyperintense signal on the axial view. MRI: magnetic resonance imaging

Given evidence of muscle inflammation, cardiac involvement, and neuromuscular weakness in the setting of recent ICI therapy, the neurology and hematology/oncology services raised concern for immune checkpoint inhibitor-related triple M overlap syndrome with immune-mediated myositis, myocarditis, and myasthenia gravis-like features. The clinical presentation was attributed primarily to ICI toxicity from pembrolizumab, as neuromuscular and cardiac immune-mediated toxicities are characteristic of ICI therapy rather than TKI therapy; however, lenvatinib has been associated with myalgia and muscle-related adverse events, and the combination of PD-1 inhibitors with TKIs may potentiate immune-related toxicities through mechanisms beyond those of PD-1 inhibition alone [11,12]. The myasthenia gravis-like component was diagnosed based on the following clinical features, which were distinguished from myositis-related weakness: bulbar dysfunction (dysphagia with nasal regurgitation, dysphonia with hypernasal/dysarthric speech), bilateral cranial nerve XI weakness (shoulder shrug), and subsequent respiratory muscle weakness culminating in respiratory failure. These features are recognized as defining characteristics of ICI-related myasthenia gravis-like syndrome in national guidelines [8]. Per recent expert consensus diagnostic criteria, a "definite" diagnosis of ICI-related myasthenia gravis requires both antibody positivity (AChR or MuSK) and electrodiagnostic evidence of a primary disorder of neuromuscular transmission; in the absence of these, the term "myasthenia gravis-like syndrome" is appropriate [9]. In this patient, serologic testing was negative for AChR and MuSK antibodies, and repetitive nerve stimulation of the ulnar and facial nerves did not demonstrate a definite decremental response; however, testing of the spinal accessory nerve was unreliable due to patient movement, and the study's sensitivity was limited by the inability to test clinically affected proximal muscles. Anti-striational antibodies (anti-titin, anti-ryanodine receptor) were not tested. Serologic testing was also negative for myositis-specific antibodies. Given the clinical, laboratory, and imaging findings consistent with ICI-related myositis, muscle biopsy was deferred based on clinical judgment that the multimodal diagnostic data were sufficient to support the diagnosis.

High-dose intravenous methylprednisolone 1 g daily was initiated, as well as intravenous immunoglobulin (IVIG) at 0.4 g/kg for a 5-day total course. Pyridostigmine was initiated per neurology recommendation and continued throughout the hospitalization. The patient was transferred to the intensive care unit (ICU) for closer monitoring. In the ICU, methylprednisolone 1 g daily was continued for a 5-day total course, followed by 1 mg/kg daily. Bedside negative inspiratory force (NIF) was measured at -20 cmH₂O, consistent with severe respiratory muscle weakness. Bedside spirometry for forced vital capacity (FVC) was not performed. Despite initial hemodynamic and respiratory stability on room air, the patient acutely decompensated following worsening hypoxemia and respiratory distress attributed to respiratory muscle weakness, requiring intubation and vasopressor support. Transthoracic echocardiography demonstrated new regional wall motion abnormalities with reduced left ventricular ejection fraction (35%), and cardiac MRI confirmed left ventricular systolic dysfunction with elevated extracellular volume fraction (ECV 32%, elevated per site-specific reference range), supporting a diagnosis of ICI-related myocarditis as seen in Figure 3. Electromyography revealed a myopathic process, with an increased proportion of short-duration, low-amplitude motor unit potentials in the deltoid and biceps brachii, as seen in Table 2.

**Figure 3:**
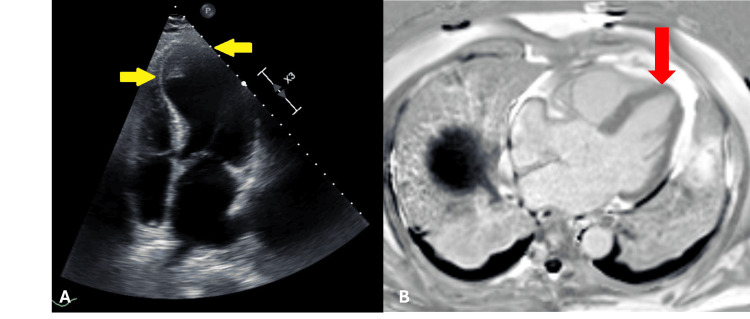
Cardiac imaging supporting the diagnosis of immune checkpoint inhibitor-related myocarditis (A) Transthoracic echocardiogram, apical four-chamber view, demonstrating severe hypokinesis of the mid and distal left ventricular segments (yellow arrows) with reduced LVEF (35%). (B) Cardiac MRI, axial view, demonstrating left ventricular myocardial hypokinesis (red arrow) with reduced LVEF and elevated extracellular volume fraction (ECV 32%). LVEF: left ventricular ejection fraction; ECV: extracellular volume fraction

**Table 2:** EMG summary table EMG results demonstrating myopathic motor unit action potential abnormalities in the right deltoid and biceps brachii, characterized by reduced amplitude and duration with increased polyphasia, consistent with an inflammatory myopathic process. EMG: electromyography Grading scale: N = normal; -1 = mildly reduced; +1 = mildly increased

	Motor unit action potential (MUAP)				
Muscle	Effort	Recruitment	Amplitude	Duration	Polyphasia
Right deltoid	N	N	N	-1	1
Right biceps brachii	N	N	-1	-1	1
Right vastus lateralis	Patient refused				

Following decompensation, the patient was transferred to a tertiary care center for consideration of plasmapheresis. On arrival, he remained intubated and hypotensive on vasopressors. Methylprednisolone and IVIG were continued. Although plasmapheresis was planned after completion of IVIG, it could not be initiated due to ongoing hemodynamic instability. The rheumatology service was consulted regarding additional immunosuppressive therapy for TMOS; however, he was deemed too unstable for further escalation of immunosuppression. His hospital course was further complicated by septic shock secondary to ventilator-associated pneumonia, acute kidney injury requiring sustained low-efficiency dialysis, and progressive acute respiratory distress syndrome (ARDS). CK and high-sensitivity cardiac troponin I levels improved significantly, as shown in Figure 4; however, with refractory ARDS and multiorgan failure, goals of care were transitioned to comfort-focused measures, and the patient died after terminal wean, as shown in Figure 5.

**Figure 4:**
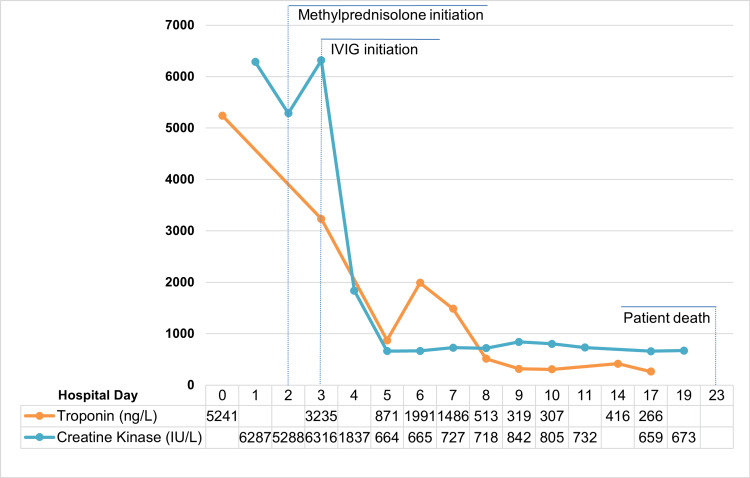
Cardiac and skeletal muscle biomarker trend during treatment Serial serum high-sensitivity cardiac troponin I and creatine kinase levels are shown over the hospital course, demonstrating sustained biomarker improvement following initiation of intravenous methylprednisolone (day 2) and IVIG (day 3), despite progressive clinical deterioration and death on hospital day 23. IVIG: intravenous immunoglobulin

**Figure 5:**
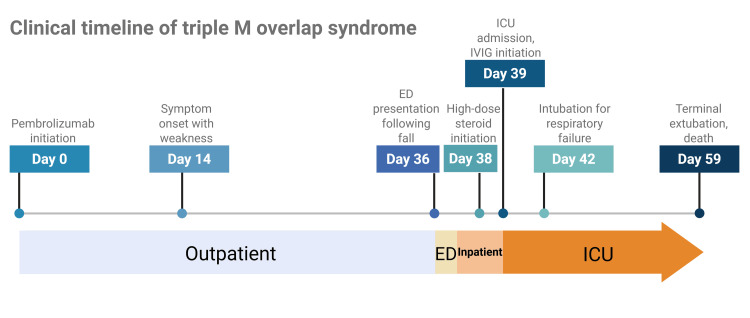
Clinical timeline summarizing key clinical events from pembrolizumab administration to patient death ED: emergency department; ICU: intensive care unit Image credit: Figure created in BioRender. Garrett, S. (2026); https://BioRender.com/80radm0

## Discussion

This case highlights the complex considerations in the management of TMOS in the setting of ICI use. Despite the great strides that ICIs have enabled in oncology, severe cardiac and neuromuscular irAEs remain a threat. Figure 6 illustrates the proposed pathophysiologic mechanisms underlying TMOS, including autoreactive T-cell-mediated inflammation, cytokine-mediated inflammation, and autoantibody production [3-5]. Shared T-cell clones targeting homologous antigens, such as α-myosin heavy chain, expressed in tumor, skeletal muscle, and cardiac muscle, are thought to drive concurrent tissue injury across these compartments [3-5,9]. Anti-striational antibodies (anti-titin, anti-ryanodine receptor) have been identified in up to 50% of ICI-related overlap cases, further supporting a shared antigenic basis for the simultaneous involvement of skeletal muscle, myocardium, and the neuromuscular junction [5,13]. Respiratory muscle involvement is a critical prognostic factor; one study showed 79% of patients with TMOS developing respiratory failure due to neuromuscular weakness, and all 8 deaths in another series examining ICI-related myositis occurred in patients with overlap syndrome involving myasthenia gravis or myocarditis [6,14]. In this patient, biomarker improvement did not translate into clinical recovery, as shown in Figure 4. It is important to note that the declining CK and troponin levels likely reflected treatment response in the cardiac and skeletal muscle compartments, while the patient's terminal trajectory was driven by complications, refractory ARDS, septic shock from ventilator-associated pneumonia, and ventilator dependence, factors that these biomarkers do not capture. Recognizing TMOS early and initiating immunosuppression are critical; however, therapeutic options are often limited by concurrent organ dysfunction and the delayed onset of action of potential treatments.

**Figure 6:**
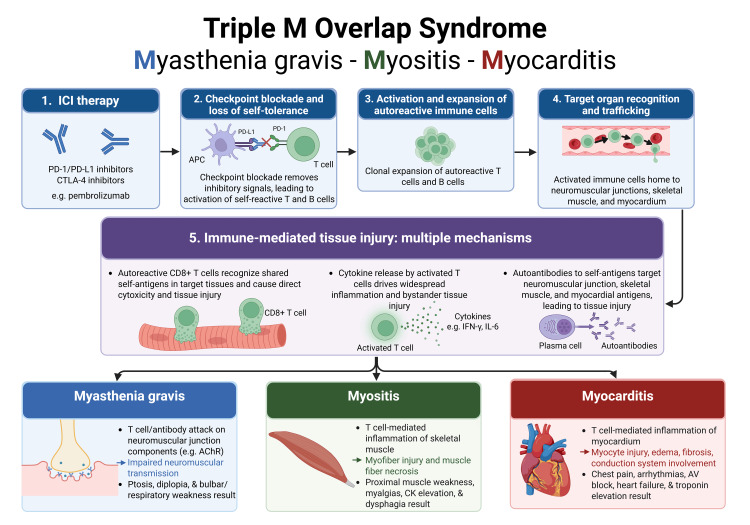
Pathophysiologic schematic of triple M overlap syndrome APC: antigen-presenting cell; AChR: acetylcholine receptor; AV: atrioventricular; CK: creatine kinase; CTLA-4: cytotoxic T lymphocyte-associated protein 4; IFN-γ: interferon gamma; IL-6: interleukin 6; PD-1: programmed cell death 1; PD-L1: programmed death-ligand 1 Image credit: Figure created in BioRender. Garrett, S. (2026) https://BioRender.com/plvgri3

Therapeutic agents for TMOS are numerous, though first-line therapy for severe irAEs, such as in this case, is high-dose corticosteroid therapy [8]. For severe myositis without improvement within 24-48 hours, guidelines recommend escalation to IVIG, plasmapheresis, or rituximab [8]. Plasmapheresis or IVIG is also recommended as initial therapy alongside corticosteroids for grade 3-4 myasthenia gravis, and both are listed among steroid-refractory options for myocarditis [8]. In cases of steroid-refractory disease, additional immunosuppressive therapy is needed. Conventional steroid-sparing medications that suppress lymphocyte proliferation, such as azathioprine and mycophenolate mofetil, are often used in the treatment of idiopathic inflammatory myopathies as well as myasthenia gravis, and their use has been extended to ICI-related disease [15,16]. However, they are limited in fulminant presentations by their delayed onset of action, typically requiring 4-12 weeks to be efficacious, a timeframe that critically ill patients may not survive. Methotrexate exerts its effects through the suppression of activated T-cell proliferation and is listed in treatment guidelines for ICI-related myocarditis and myositis that are steroid-refractory; however, its use here is similarly limited by prolonged time to efficacy as well as hepatotoxicity [8].

Abatacept, which blocks T-cell co-stimulation driving the fulminant inflammatory response in ICI-induced myocarditis, was utilized in an earlier case report demonstrating complete resolution of severe, steroid-refractory ICI-induced myocarditis with features of myositis and myasthenia gravis-like syndrome [17]. Subsequently, reversal of a severe case was demonstrated by using high-dose, personalized dosing targeting >80% CD86 receptor occupancy, combined with ruxolitinib and methylprednisolone [18]. In a prospective observational study by Salem et al., early initiation of abatacept and ruxolitinib combined with systematic screening for respiratory muscle involvement was associated with a myotoxicity-related fatality rate of 3.4% (1/30) compared to 60% in a historical control group receiving conventional high-dose steroid therapy alone (p<0.0001) [19]. The ACHLYS trial is an ongoing phase II dose-finding trial evaluating abatacept for ICI-related myocarditis, in combination with corticosteroids and ruxolitinib, and this regimen is already acknowledged in national guidelines as having been used in clinical practice [8,20]. A 2025 American College of Cardiology Concise Clinical Guidance also notes that targeted immunomodulatory approaches with abatacept and ruxolitinib upfront may have fewer side effects than conventional high-dose steroid therapy alone [21]. Although its mechanism of inducing global T-cell anergy is well-suited for reversing ICI-activated pathways, the time to clinical efficacy and increased risk of infection limit its availability as an option for patients with septic shock and multiorgan failure.

Pyridostigmine, a standard symptomatic treatment for myasthenia gravis, was initiated per neurology recommendations and continued throughout the hospitalization. Notably, national guidelines list pyridostigmine as a treatment option for grade 2 ICI-related myasthenia gravis but do not include it in the grade 3-4 management algorithm, and advise avoiding medications that can worsen myasthenia, including beta-blockers, fluoroquinolones, and IV magnesium in severe disease [8]. Its use in the setting of concurrent myocarditis warrants caution due to potential cholinergic effects on cardiac conduction, though it was continued in this case without apparent cardiac worsening. A recent multicenter comparative study found that pyridostigmine provided clinical benefit in only 61.5% of ICI-related myasthenia gravis-myositis overlap cases compared to 94.4% in sporadic cases, suggesting that the neuromuscular junction component may be less responsive to acetylcholinesterase inhibition in the ICI setting [10].

Rituximab could have provided significant benefit in this case through B-cell depletion, thereby targeting antigen presentation, autoantibody production, and ultimately the cross-talk between B and T lymphocytes, common mechanisms for both ICI-related myositis and myasthenia gravis [22]. Given its demonstrated efficacy for both conditions, it is currently listed in national guidelines as a treatment option for ICI-related myasthenia gravis when refractory to plasmapheresis or IVIG, and for severe or life-threatening myositis refractory to steroids with minimal improvement after 24-48 hours [8]. A recent multicenter retrospective study of rituximab for severe ICI-related irAEs (n=18, including 6 with myositis and myocarditis) demonstrated clinical improvement in 83% of patients, including complete resolution in 39% [23]. A case of nivolumab- and ipilimumab-induced triple overlap syndrome successfully treated with rituximab after failure of methylprednisolone, IVIG, and plasma exchange has also been reported [24]. Its faster onset of B-cell depletion compared to conventional steroid-sparing agents would make it a therapeutically advantageous option, particularly when combined with plasmapheresis. Given the patient's hemodynamic instability, this option could not be initiated safely, highlighting another therapeutic limitation in this case.

IL-6 receptor antagonists, such as tocilizumab, have shown promising preliminary results in irAE treatment. In a conference abstract, Obeid et al. reported that all studied cases of steroid-refractory myocarditis (n=8), including those unresponsive to mycophenolate mofetil or infliximab, responded effectively to tocilizumab [25]. However, as this evidence is derived from a conference abstract rather than a peer-reviewed publication, it should be interpreted with appropriate caution. Furthermore, the hepatotoxicity risks of tocilizumab contraindicate its use in this patient with pre-existing drug-induced liver injury from lenvatinib.

This case differs from previously reported TMOS cases in a critical respect. In prior reports, escalation of immunosuppression, whether with abatacept, rituximab, tocilizumab, or plasmapheresis, was feasible and often led to clinical improvement [17-19,23,24]. In contrast, this case illustrates a scenario in which the multiorgan injury inherent to TMOS, compounded by TKI-related hepatotoxicity from lenvatinib, progressively eliminated each available therapeutic option, creating a self-reinforcing cycle of therapeutic limitation. Lenvatinib-related hepatotoxicity, a well-documented adverse effect occurring in over 50% of patients receiving the pembrolizumab-lenvatinib combination, was already present and worsening at the time of TMOS diagnosis (alanine aminotransferase (ALT) 800 IU/L, aspartate aminotransferase (AST) 560 IU/L), establishing a baseline hepatic injury that immediately constrained the available therapeutic options [12,26]. Hepatotoxicity contraindicated tocilizumab and methotrexate; hemodynamic instability precluded plasmapheresis, rituximab, and further escalation of immunosuppression; and the delayed onset of action of conventional steroid-sparing agents (azathioprine, mycophenolate mofetil) rendered them impractical in this fulminant presentation. This structural vulnerability of TMOS management, in which the syndrome's own multiorgan injury narrows the treatment window, should be anticipated in any severe case, particularly when concurrent TKI-related toxicity is present.

Despite the comprehensive investigation involved in diagnosing TMOS in this patient, several limitations warrant discussion. First, a muscle biopsy was not performed; histopathologic confirmation (CD8+ T-cell infiltrates, necrosis, MHC-I upregulation) could have added further diagnostic certainty and differentiated ICI-related inflammatory myopathy from drug-induced toxic myopathy from lenvatinib - a distinction that would have been clinically relevant for guiding therapeutic decisions, though in this case, it was apparent his complications were consistent with TMOS in the setting of ICI use [5]. Second, the myasthenia gravis-like component was diagnosed clinically, as repetitive nerve stimulation was limited and restricted in scope due to patient intolerance, and serologic testing for AChR and MuSK antibodies was negative. Anti-striational antibodies (anti-titin, anti-ryanodine receptor) were not tested. Recent evidence suggests that objective neuromuscular junction dysfunction may be limited even in anti-AChR-positive ICI-related overlap patients, and that myasthenia gravis may be overdiagnosed in the ICI setting, with clinically reported fatigue potentially reflecting reduced muscle endurance from inflammatory myopathy rather than true fatigability from primary neuromuscular junction dysfunction [10]. The absence of formal bedside fatigability testing is a limitation; however, the severity of fixed proximal weakness at presentation would have limited the interpretability of such testing. Notably, the lack of associated fatigable weakness in ICI myopathy has been identified as a feature that assists in differentiation from myasthenia gravis [9,10]. Third, LDH, aldolase, ESR, CRP, and ferritin were not obtained on admission, limiting the baseline inflammatory and muscle injury assessment. Fourth, while TMOS was the initiating cause of the patient's deterioration, the terminal events were substantially driven by complications of mechanical ventilation and immunosuppression, specifically septic shock from ventilator-associated pneumonia and refractory ARDS, rather than by TMOS-intrinsic organ injury alone. However, the clinical timeline and multimodal diagnostic data are consistent with TMOS as the patient's clinical syndrome secondary to pembrolizumab administration.

## Conclusions

In conclusion, TMOS often presents a challenging clinical syndrome to manage. Its propensity to cause multisystem dysfunction can be directly life-threatening, while also precipitating complications such as hemodynamic instability, organ failure, and increased infection risk from iatrogenic immunosuppression that create contraindications to the very immunosuppressive therapies required for definitive treatment. As a single case, generalizability is inherently limited; however, this structural vulnerability of TMOS management should be anticipated in any severe case, particularly when concurrent TKI-related hepatotoxicity is present. While many of the effective therapeutic agents, such as conventional steroid-sparing agents and abatacept, may be appropriate for cases with less immediately life-threatening features and more indolent courses, rapidly progressive cases may warrant early consideration of rituximab in combination with plasmapheresis. This recommendation is based on mechanistic rationale and limited case series rather than controlled evidence, and should be considered expert opinion pending further study. This self-reinforcing cycle of therapeutic limitation highlights the importance of having a low threshold for suspicion of TMOS and an aggressive management plan prior to end-organ damage, narrowing the treatment window. As demonstrated in this case, biomarker improvement alone is inadequate to assess clinical prognosis, as the declining CK and troponin likely reflected treatment response in the cardiac and skeletal muscle compartments, while the terminal trajectory was driven by ARDS, septic shock, and ventilator dependence. Therefore, early recognition and rapid escalation of immunosuppression are critical in TMOS due to its high mortality.
